# Editorial

**Published:** 1843-11-25

**Authors:** 


### THE MEDICAL EXAMINER.

PHILADELPHIA, NOV. 25, 1843.

In the 20th number of this Journal an article appeared in the "Retrospect," copied from a late number of the London Medical Gazette, and written by Dr. Knox, of Edinburgh, the object of which was to deny the claims of Professor Horner, of this city, to the discovery of the Tensor Tarsi muscle, and to 'restore' it to Duverney. A severe scrutiny of the claims of our countryman took place in Europe on the announcement of the discovery in 1824, and the discovery conceded to him. The tensor tarsi muscle, as described by Dr. Horner, lies on the posterior face of lacrymal sac and ducts; it is oblong; and in the adult, is three lines broad and six long. It arises from the posterior, superior face of the os unguis, and running forward, is inserted by a bifurcated termination into the puncta lachrymalia. Duverney's muscle arises from the os planum, and is inserted into the tendon of the orbicularis. Rosenmuller's description, mentioned by Dr. Knox, is a repetition of Duverney's. Professor Horner's claims are, we believe, now universally admitted by all anatomists of note, amongst others we may mention Theile, one of the latest and best authorities, who was especially selected to furnish the volume on the muscles to the new Anatomical Encyclopædia, now pasing through the German press.

